# Acute Endothelial Benefits of Fat Restriction over Carbohydrate Restriction in Type 2 Diabetes Mellitus: Beyond Carbs and Fats

**DOI:** 10.3390/nu10121859

**Published:** 2018-12-01

**Authors:** Renate Luzía Barbosa-Yañez, Ulrike Dambeck, Linna Li, Jürgen Machann, Stefan Kabisch, Andreas F.H. Pfeiffer

**Affiliations:** 1 Department of Clinical Nutrition, German Institute of Human Nutrition Potsdam-Rehbruecke, 14558 Nuthetal, Germany; ulrikedambeck@gmx.de (U.D.); stefan.kabisch@dife.de (S.K.); 2 German Center for Diabetes Research (Deutsches Zentrum für Diabetesforschung e.V.), Ingolstädter Landstraße 1, 85764 Neuherberg, Germany; Juergen.Machann@med.uni-tuebingen.de; 3 Department of Endocrinology, Diabetes and Nutrition, Campus Benjamin Franklin, Charité University Medicine, Hindenburgdamm 30, 12203 Berlin, Germany; Linna.li@charite.de; 4 Institute of Diabetes Research and Metabolic Diseases of the Helmholtz Center Munich at the University of Tübingen, 72076 Tübingen, Germany; 5 Section on Experimental Radiology, Department of Diagnostic and Interventional Radiology, University Hospital Tübingen, 72076 Tübingen, Germany

**Keywords:** type 2 diabetes, endothelial function, intrahepatic lipids, low fat diet, low carbohydrate diet, protein intake

## Abstract

Background: Cardiovascular diseases (CVD) are the major cause of mortality in type 2 diabetes patients (T2DM). The causes are embedded in a complex interplay between excess body fat, insulin resistance and serum lipid anomalies. Endothelial homeostasis is strongly affected by this pathogenic network. Even though metabolic changes and weight loss improve vascular endothelial function, the effect of different dietary approaches is still uncertain for type 2 diabetes patients. Objective: We aimed to compare the acute effects of a hypocaloric very low carbohydrate (VLC) diet versus a hypocaloric low fat (LF) diet on flow mediated dilation (FMD), intrahepatic lipid (IHL) accumulation and visceral adipose tissue as independent risk factors of CVD in T2DM patients. Design: 36 T2DM patients (age 63 ± 8 years, 60% females) were randomly assigned to the VLC diet (4–10% of total energy intake (E)) or to the LF diet (<30% E) for 3 weeks. Endothelial function was assessed by the flow mediated dilation (FMD) method. Adipose tissue depots and IHL were determined by magnetic resonance. Results: Both dietary strategies reduced body weight, body fat content and IHL. Unexpectedly, the LF group experienced significantly greater enhancement of FMD, compared to the VLC group. The FMD showed a positive correlation with protein intake and fat intake in the LF group, while it revealed a negative correlation with protein intake in the VLC diet group. Conclusions: Reduction of total and hepatic adiposity was shown to be successful using either the VLC or LF hypocaloric diets, however, improvements in FMD may be related to the interplay of fat and protein intake.

## 1. Introduction

The control of Type 2 diabetes mellitus (T2DM) has become a global challenge. Its incidence, prevalence and costs are rapidly increasing often in parallel with obesity, cardiovascular diseases (CVD) and nonalcoholic fatty liver disease (NAFLD), embracing a dysfunctional metabolic network. Excess body weight seems to be the main driver of this pathogenic network and probably provides the common link between T2DM, NAFLD and CVD through insulin resistance, hyperlipidemia, hypertension and low-grade inflammation [1,2]. Given that CVD is the major cause of mortality in T2DM [3], the impact of this dysfunctional network on vascular events needs to be considered. It is well established that vascular endothelial cells play a pivotal role in the maintenance of cardiovascular homeostasis. The endothelium accounts with two main mechanisms to control the internal stability: Vasodilation and vasoconstriction [4]. Perhaps the most important mechanism for endothelial relaxation in arteries is the endothelium-dependent synthesis of nitric oxide (NO), an effective vasodilator and a functional marker of endothelial dysfunction [4]. In fact, endothelial dysfunction has been associated with T2DM [5], NAFLD [6] and excessive fat mass [7].

With a non-invasive methodology it is possible to quantify the ability of the brachial artery to dilate in response to shear stress [8]. The Flow Mediated Dilation (FMD) technique has been applied in several clinical studies to determine the effect of weight loss (by hypocaloric diets, bariatric surgery or/and exercise) [9,10,11,12,13], low fat (LF) and low carbohydrate (LC) diets [14,15,16,17,18,19] on vascular function. Indeed, weight loss is a key determinant for the restoration of vessel stability and integrity, but also the reduction of visceral fat [20], liver fat [21] and glycated hemoglobin A1c (HbA1c) levels [22] contribute to an enhanced FMD.

Hypocaloric nutritional strategies are a common tool used for T2DM management due to their rapid metabolic benefits. However, the macronutrient composition of such low caloric diets is still under dispute regarding its impact on endothelial function in T2DM patients.

Therefore, the aim of this study was to examine and compare the effect of a hypocaloric very low carbohydrate (VLC) diet versus a hypocaloric LF diet on FMD, intrahepatic lipid (IHL) accumulation and visceral adipose tissue (VAT), as independent risk factors of CVD in T2DM patients and to evaluate the macronutrient composition of each diet and its relation to FMD at intervention completion.

## 2. Materials and Methods

### 2.1. Participants and Study Design

The DiNA-D (Diabetes Nutrition Algorithms in Patients with Overt Diabetes Mellitus) study was a randomized, parallel group, intervention study with adult T2DM patients. Following the approval of the Ethics Committee of the Charité and of the University of Potsdam, conducted in accordance with the Declaration of Helsinki, the study was registered at www.ClinicalTrials.gov (NCT02459496). The study was conducted at the clinical ward in Berlin, Germany (located at the Charité Campus Benjamin Franklin). Study participants were recruited via local advertisements. Volunteers, who met the inclusion criteria, provided written informed consent according to Good Clinical Practice (GCP) guidelines. After the initial screening (V0) subjects were randomized according to a 1:1 ratio based on either a VLC or LF diet using computer generated random numbers and stratified according to age, sex, body mass index (BMI) and waist circumference. The subjects visited the study facility twice, at baseline (V1) and post-intervention (V2) of the respective dietary intervention, where routine clinical measurements and MRI/1H-MRS (magnetic resonance imaging/magnetic resonance spectroscopy) (Siemens Healthineers, Erlangen, Germany) assessments were performed.

This paper reports the outcomes of the study subgroup with an assessment of the endothelial function, over a 3 week period, conducted between October 2013 and February 2016.

### 2.2. Dietary Intervention

The VLC diet was aimed to be ketogenic with a carbohydrate daily intake of ˂40 g (60–70% fat, 5–10% carbohydrate, 20–30% protein) and included a reduction of caloric intake to 1200–1500 kcal/day. Study participants received recipes, daily meal plans and a substitution list with common foods to restrict carbohydrates and calories intake.

The LF was characterized by a calorie intake of 1000–1200 kcal/day, and less than 30% of the total energy intake (E%) of fat (<30% fat, 50% carbohydrate, 20% protein). Study participants were provided with a flavored meal replacement powder (MODIFAST^®^ (OTC Siebenhandl GmbH, Ulm, Germany)) (Supplementary Materials Table S1). In addition, the intake of 200 g of raw or steamed vegetables was allowed.

### 2.3. Body Weight and Body Composition

Weight and height were determined with the electronic weighing and measuring station seca 764 (Approval class III, Seca Ltd., Birmingham, England).

### 2.4. Liver Fat and Visceral Fat

Magnetic resonance for the determination of IHL and adipose tissue compartments were performed under fasting conditions as described in [23]. Briefly, IHL was quantified by proton magnetic resonance spectroscopy (1H-MRS) on a 1.5 T Magnetom Avanto (Siemens Healthineers, Erlangen, Germany) applying a single voxel STEAM (Stimulated Echo Acquisition Mode) technique (TE (echo time) = 10 ms, TR (repetition time) = 4 s). Abdominal adipose tissue was quantified from axial T1-weighted fast-spin echo images between the hip and shoulder with subjects lying in prone position with outstretched arms. Total abdominal adipose tissue (TAT) and VAT depots were quantified by an automatic segmentation procedure based on fuzzy clustering and orthonormal snakes [24].

### 2.5. Clinical Parameters

Levels of HbA1c were determined in the fasting state in serum/plasma with ABX Pentra 400 (Horiba, Fukuoka, Japan). For determining the blood levels of insulin (Mercodia, Uppsala, Sweden), an enzyme-linked immunosorbent assay was used. Glucose and CRP (c-reactive protein) was determined by turbidimetric immunoprecipitation.

A fasting blood sample was collected at baseline and post-intervention diet; serum lipids (total cholesterol (TCHO), high density cholesterol (HDL), low density cholesterol (LDL), triglycerides (TAG) and further routine parameters were measured in serum/plasma by using ABX Pentra 400 (Horiba, Fukuoka, Japan).

All measurements of systolic blood pressure (SBP) and diastolic blood pressure (DBP) were performed after a previous resting time of 10 min at room temperature using a standardized blood pressure cuff system (BOSO ABI system 100, BOSO, Jungingen, Germany).

### 2.6. Endothelial Function

All measurements were performed by an experienced member of staff, according to a standardized protocol in the morning. Furthermore, participants were asked to pause taking their antidiabetic medications 3 days prior to the measurement and to avoid the consumption of alcohol, caffeine and tobacco for 8 h before the measurement.

Brachial artery ultrasound was performed in the fasted state using a 12-MHz probe and high resolution ultrasound. Ultrasound images were video recorded and digitalized using a highly sophisticated computerized system to detect the intima media complex by edge detection software (Brachial Analyzer version 5.10.6, Medical Imaging Applications LLC, Iowa City, IA, USA).

Participants were asked to rest quietly for 10 min before the measurement started. In this time a sphygmomanometric cuff was placed around the right arm just above the medial epicondyle of the humerus and identification of the endothelium was performed. A longitudinal image of the artery anterior and posterior vessel wall was visualized, the subject’s arm was held steady and the resting baseline images of the brachial artery diameter were video recorded for 5 s in B-Mode [8]. Thereafter, the volumetric blood flow measurement was performed using the PW-Doppler mode. Arterial occlusion was then generated by inflating the sphygmomanometric cuff on the right arm to 260 mmHg for 1 min and retained for 5 min. The post occlusion dilation of the artery was video recorded for 5 min. During the depressurization, the increase in flow velocity was also recorded and a 5 s sequence was saved in B mode within the following 15 s. In the time interval of 4 min after the cuff release, a video sequence was recorded every 15 s at the beginning and every 30 s during the last minute in the B-screen. Besides the initial diameter, the shear stress mediated dilatation was recorded at 14 time points. A video sequence entailed 94 single images and thus provided 94 values of the luminal diameter. For the calculation of the FMD, values were averaged and from a total of 14 time points, the peak diameters were used.

### 2.7. Diet Compliance and Evaluation

To ensure compliance, participants of both groups were given dietary counseling and support at baseline by a certified nutritionist and during the diet phase, telephone follow up was carried out. Furthermore, all subjects were asked to document their food intake throughout the intervention (21 days). Diet records were analyzed for macronutrient content using Prodi software (Version 6.2, Nutri-Science, Hausach, Germany). A mean value of 4 days before starting the diet was calculated for the dietary intake at baseline. A mean value of 3 weeks diet (21 days) was calculated for the dietary intake at endpoint.

### 2.8. Statistical Analysis

Statistical analysis was conducted using the SPSS software package for Windows (IBM, version 20.0, Chicago, IL, USA). The results were expressed as mean ± SD. After a plausibility assessment, extreme values of FMD were excluded from analysis: Two study participants, one of each diet group, had FMD values beyond the outer borders of the boxplot analysis, and therefore were considered extreme outliers. Data were examined for normality by the KolmogoroV–SmirnoV test. For comparison within and between diet groups, a Student’s *t*-test (paired and unpaired) was performed. Non-normally distributed variables were analyzed by non-parametric tests (Wilcoxon or Mann–Whitney-U-Test). FMD was separately analyzed by Analysis of variance (ANOVA) for repeated measurements for assessing diet-FMD interaction effects. A model with one between- subject factor (diet groups) and two within-subject factors (V1 and V2) was used and was adjusted for kilocalories intake change per kg BW.

Pearson correlation analysis was performed to identify the strength of relations between macronutrients intake (g) per kg body weight (BW) and FMD after dietary intervention. Non-normally distributed variables were analyzed by Spearman correlation analysis. Statistical significance was defined as *p* < 0.05.

## 3. Results

**Figure 1:**
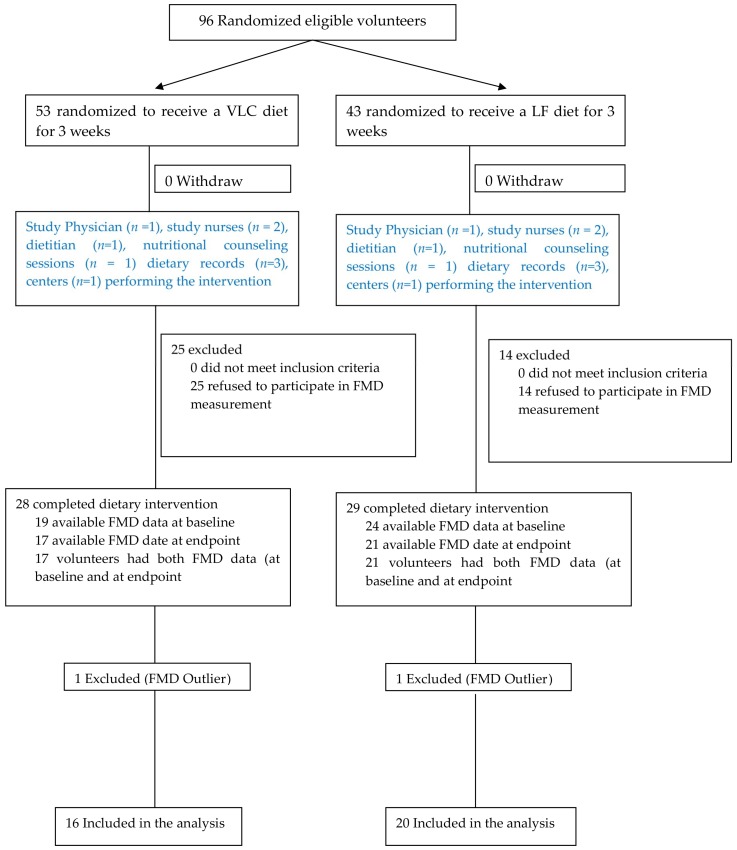
Flow diagram of the subjects included in this study. Adapted version of “CONSORT flow diagram for individual randomized controlled trials of nonpharmacological treatments” [26] illustrating participants inclusion to the FMD sub-group. FMD: Flow mediated dilation; VLC: Very low carbohydrate diet; LF: Low fat diet.

A total of 36 T2DM patients completed the intervention study (Figure 1). Participants consisted of 22 females and 15 males, on average 63 years of age (range: 42–76 years), were mostly overweight to mildly obese (mean BMI = 33 ± 5 kg/m^2^) with a mean HbA1c level of 6.5 ± 0.8% (47 ± 8 mmol/mol). Furthermore, 64% of all participants showed a liver fat content above the cutoff value of 5.56% for the clinical diagnosis of NAFLD [25] (mean IHL = 12.9 ± 9.5 %). About 86 % were non-smokers or former smokers (Table 1).

**Figure 2:**
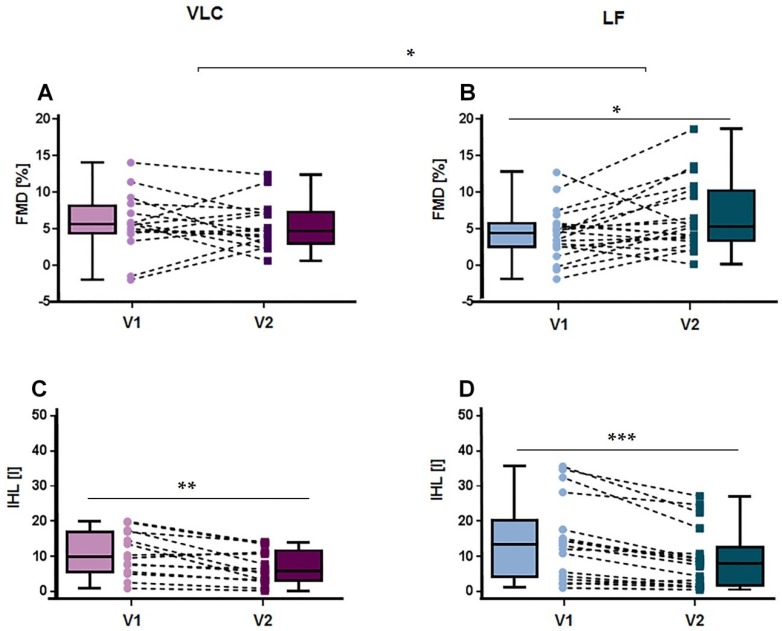
Acute effects on FMD and IHL after 3 weeks diet. Boxplots and individual changes in endothelial function (**A**) and intra hepatic lipids (**C**) post-intervention on the very low carbohydrate (VLC) diet; changes in endothelial function (**B**) and intra hepatic lipids (**D**) after 3 weeks on the low fat (LF) diet. FMD = flow mediated dilation, IHL = intra hepatic lipids, N_VLC_ = 16, N_LF_ = 20, V1 = Visit 1, V2 = Visit 2, * *p* < 0.05; ** *p* < 0.01; *** *p* < 0.001.

Table 2 displays results of anthropometric differences within and between diet groups. In brief, after 3 weeks of dietary intervention, both the LF and the VLC diet significantly reduced body weight by −4.1 kg and −5.2 kg, respectively (*p*_VLC_ < 0.001, *p*_LF_ < 0.001). Similarly, body fat content decreased significantly in different compartments in both diet groups (TAT (*p*_VLC_ = 0.001, *p*_LF_ < 0.001), VAT (*p*_TAT_ = 0.024, *p*_VAT_ < 0.001) and IHL content decreased extensively by 35–36% relative to the initial levels (*p*_VLC_ = 0.003, *p*_LF_ < 0.001)) (Figure 2).

However, HbA1c levels declined significantly only in the VLC group (*p*_VLC_ < 0.001, *p*_LF_ = 0.054, *p*_Δ_ = 0.001). No further significant differences were found between diet groups in any of these variables. Despite the fact that both dietary approaches showed similar improvements in total cholesterol (*p*_VLC_ = 0.001, *p*_LF_ < 0.001), LDL (*p*_VLC_ = 0.004, *p*_LF_ < 0.001) and TAG (*p*_VLC_ = 0.003, *p*_LF_ = 0.042), LDL (*p*_V2_ = 0.035) and total cholesterol (*p*_V2_ = 0.042) had significantly greater decreases in the LF compared to the VLC diet.

Further significant improvements were observed for SBP, (*p*_VLC_ = 0.003, *p*_LF_ = 0.042) (Table 3).

FMD showed no significant differences between groups at baseline (*p*_FMD_ = 0.267) and did not change significantly in the VLC diet group (*p*_VLC_ = 0.782) post-intervention (Figure 2). However, after the LF diet FMD increased significantly (*p*_LF_ = 0.024) (Figure 2).

Nutritional data were adjusted for kg BW (past studies have proposed that weight loss [10,11] is effective in improving flow mediated vasodilation). For this reason, macronutrients intake was corrected for weight (kg/BW). The intake of energy (*p*_VLC_ < 0.001, *p*_LF_ < 0.001, *p*_Δ_ < 0.001, *p*_V2_ = 0.001), protein (*p*_VLC_ = 0.002, *p*_LF_ < 0.001, *p*_Δ_ < 0.001, *p*_V2_ < 0.001) and carbohydrate (*p*_VLC_ < 0.001, *p*_LF_ = 0.006, *p*_Δ_ = 0.005, *p*_V2_ < 0.001) showed significant differences within and between diet groups (Table 4). Total fat intake showed no significant changes in the VLC diet, while, as expected, decreased significantly in the LF group (*p*_LF_ < 0.001), resulting in significant differences between diet groups (*p*_Δ_ < 0.001, *p*_V2_ < 0.001.

Accordingly, ANOVA model for FMD was adjusted for energy intake changes. Table 5 shows the significant interaction effect between diet and FMD that was found (*p*_visit_ = 0.267, *p*_visit x diet_ = 0.034) (Table 5).

**Figure 3:**
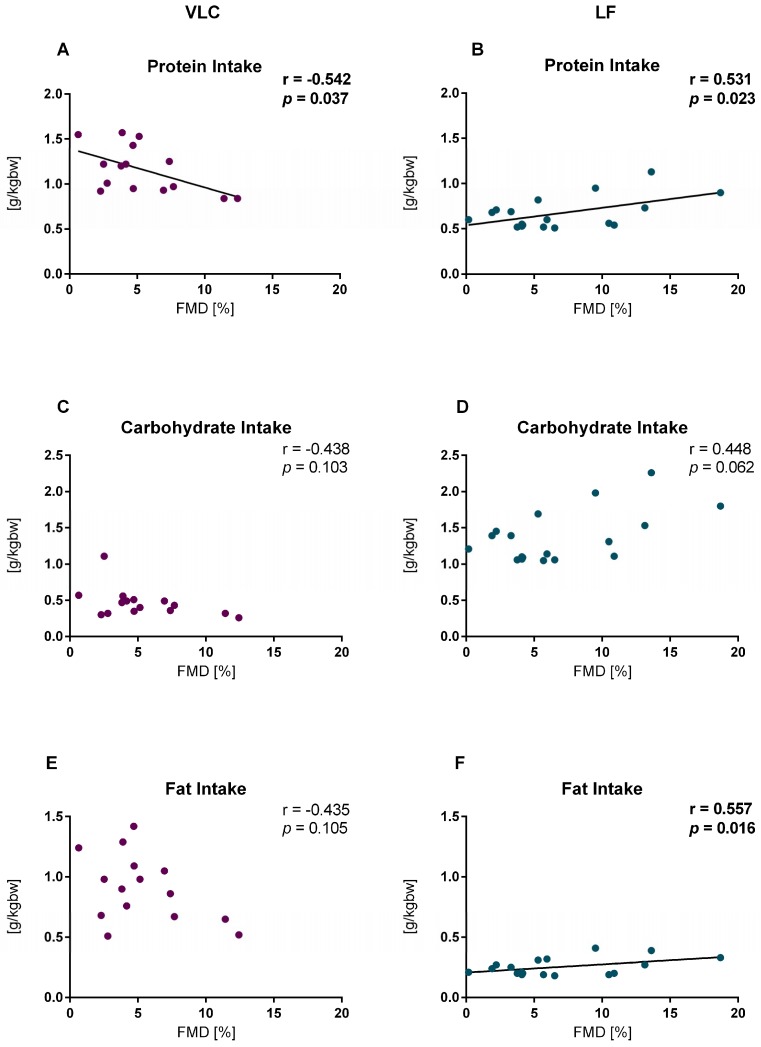
Correlations between flow mediated dilation (FMD) and macronutrient intake after 3 weeks diet. (**A**) Protein intake at Visit 2 (V2) in the very low carbohydrate (VLC) diet, *n* = 15, (**B**) protein intake at V2 in the low fat (LF) diet, *n* = 18, (**C**) carbohydrate intake at V2 in the VLC diet, *n* = 15 (**D**) carbohydrate intake at V2 in the LF diet, *n* = 18, (**E**) fat intake at V2 in the VLC diet, *n* = 15 (**F**), fat intake at V2 in the LF diet, *n* = 18. A–F show data after macronutrient intake correction for body weight at V2.

After 3 weeks on a LF diet, FMD showed a significant positive correlation with protein (g/kg BW) (*r* = 0.531, *p* = 0.023) and fat intake (g/kg BW) (*r* = 0.557, *p* = 0.016). On the contrary, the VLC group showed a significant negative correlation with protein intake (g/kg BW) (*r* = −0.542, *p* = 0.037) (Figure 3).

## 4. Discussion

In the present study we observed that both dietary strategies, VLC and LF, effectively reduced body weight, TAT, VAT and IHL. This suggested that both diets had an acute effect on the pathologic network, which has been previously associated with damaged endothelial function [5,6,7,20,21]. However, independent of the body fat decreasing success of both diets, subjects who participated in the LF diet experienced significantly greater enhancement of the ability of the brachial artery to dilate, compared to subjects on the VLC diet. We showed that the favorable endothelial nature of the LF diet mainly related to its protein quantity and source, both corresponding to fat content and its shares in saturated and unsaturated fatty acids.

After just 3 weeks on a VLC or a LF diet, subjects lost significant weight and decreased TAT, VAT, IHL, TCHO, LDL, and TAG. In spite of the fact that only the VLC diet significantly reduced HbA1c levels and in agreement with previous studies [12,13,16,17], our outcomes suggested that the LF diet had an acute vasodilation effect compared to the VLC diet, whereby the LF diet showed a significant increase of 2.27% points.

According to the evaluation of diet records, subjects of both diet groups adhered well to their respective diets. However, we detected a significant difference in energy consumption between diet groups. This may be related to the previously mentioned weight loss difference between diet groups. Subjects on the LF diet consumed 381 Kcal less than the VLC diet group, and therefore lost 1.1 kg more body weight. Consequently, macronutrient intake analysis was corrected for kg BW revealing that the VLC diet increased their protein ingestion by 0.21 g/kg BW, while the LF diet decreased it by −0.26 g/kg BW.

### 4.1. Protein Intake

Our results demonstrate that the positive FMD effect of the LF diet is partially driven by protein. Post-intervention the VLC diet showed a negative correlation and the LF diet showed a positive correlation of FMD with protein intake (g/kg BW). Certainly, protein consumption is often tied to fat consumption. Dependent on the protein source the total fat intake and ratio between fatty acids can vary. The required carbohydrate restriction of the VLC diet increased the amount of animal protein sources (meat, cheese), while the meal replacement powder of the LF diet was rich in isolated milk protein and contained defined fats. Whey protein and casein are high quality proteins based on bioavailability, digestibility, amino acid requirements and also bioactive peptides. Currently, there is substantial interest in milk proteins to improve vascular health, which has been extensively reviewed [27]. Different research groups showed this positive effect of whey protein [28,29] and casein [30] supplementation on FMD. Although the molecular mechanism behind this is still unclear, the present study corroborates that protein quality and quantity do play a part in the FMD response. However, it is very challenging to separate the effect of single macro-(micro) nutrients without reflecting the modifications of other macronutrients, since they are connected (discussed below).

### 4.2. Fat Intake

Our analysis suggests that the intake of energy restricted LF diet leads to an enhanced endothelial function attributed perhaps in part to the reduced fat composition of the LF diet. However, it seems that subjects with the lowest fat consumption had the lowest FMD, while subjects with the highest intake of fat had the highest FMD. On the contrary, in the VLC group, although the correlation was not significant, subjects with the lowest fat consumption tended to have the highest FMD and subjects with the highest intake of fat had the lowest FMD. This suggests a potential interaction of protein and fat intake. Indeed, we previously reported a key role of protein intake in modulating fat metabolism particularly with regard to lipolytic activity and modulation of free fatty acids (FFA) [23]. The *n*-3 FFA alpha-linolenic acid (ALA) and *n*-6 FFA linoleic acid compete for the delta5 and delta6 desaturases and elongase pathways resulting in the generation of either eicosapentaenoic acid or arachidonic acid. The latter is a precursor for numerous bioactive lipids of the prostanoid family with powerful endothelial activity [31]. On the other hand, the long chain *n*-3 fatty acids display anti-inflammatory properties, although the supplementation of n3 polyunsaturated fatty acids (PUFA) did not result in improved FMD in a recent study [32].

### 4.3. Carbohydrate Intake

A hand full of comparable short-term studies investigating the dietary influence on FMD have been performed. Phillips et al. [17] and Varady et al. [16] observed this endothelial “LF benefit” after 6 weeks of diet. However, both research groups also described an endothelial “VLC impairment”. Our study cannot confirm this observation, since we found no significant difference within (V1 vs V2) this diet group. Furthermore, correlation analysis between carbohydrate intake and FMD was not significant in either diet group.

In contrast to the VLC diet of the present study the high fat (VLC) diet was characterized by a carbohydrate intake of ca. 20 g/day and by a higher intake of SFA, which probably has adverse effects on endothelial function [15,18].

The outcomes with VLC diets may improve by decreasing SFA while increasing PUFA and MUFA and perhaps by introducing whey protein and casein as the main protein sources.

The main limitation of our study is that the self-reported diet records to assess food intake were a subjective tool, which may be an important confounder. Further limitations are that this study is limited to subjects of Caucasian ethnicity and the small sample size.

## 5. Conclusions

In conclusion, we demonstrate that both, VLC and LF diets are effective tools for rapid reduction of weight, TAT, VAT, IHL and blood lipids in T2DM patients. Nevertheless, the LF diet elicited advantageous effects on flow mediated arterial dilation despite the greater HbA1c decrease of the VLC diet. The potential driver of this acute endothelial response may be the interaction of protein and fat metabolism saturated fats. Identification of molecular mechanisms underlying FMD may provide a greater understanding of diet-induced effects on endothelial function.

## Figures and Tables

**Table 1:** Baseline characteristics of subjects.

General Parameters	Subset	Mean	±SD
**Age** ^1^		63	(±8)
		*N*	%
**Sex** ^2^	♀	22	60
	♂	14	40
VLC	♀	11	69
	♂	5	31
LF	♀	10	53
	♂	9	47
**Lifestyle** ^2^		*n*	(%)
Smoking Status	Never smoked	14	39
	Smoker	5	14
	Ex-smoker	17	47
**Medication** ^2^			
Oral antihyperglycemic agents	0	13	37
	1	16	45
	2	4	11
Insulin	0	30	86
	1	3	9
Lipid lowering agents	0	20	57
	1	13	37
Blood pressure lowering agents	0	6	18
	1	15	46
	2	7	21
	3	4	12
	4	1	3
Bradycardic antihypertensive agents	0	19	54
	1	12	34
	2	2	6

Values are presented as mean (±SD) ^1^ or as frequency (%) ^2^. *n* = 36. VLC: Very low carbohydrate diet, LF: Low fat diet. Medication: 1 = 1 agent, 2 = 2 different agents, 3 = 3 different agents, 4 = 4 different agents.

**Table 2:** Anthropometric differences within and between diet groups.

												V1 vs V2		VLC vs LF		
Anthropometry				VLC			Δ	LF			Δ	VLC	LF	Δ	V1	V2
			Visit	*n*	Mean	±SD		*n*	Mean	±SD		*p*-Value	*p*-Value	*p*-Value	*p*-Value	*p*-Value
Weight (kg)			V1	16	93.9	20.7	−4.1	20	97.6	22.6	−5.2	**<0.001**	**<0.001**	ns	ns	ns
			V2	16	89.8	19.7		20	92.5	20.9						
BMI (kg/m^2^)			V1	16	32.1	4.5	−1.3	20	32.7	4.9	−1.6	**<0.001**	**<0.001**	ns	ns	ns
			V2	16	30.8	4.3		20	31.1	4.6						
TAT [l]			V1	14	23.4	6.9	−1.7	18	22.9	5.3	−1.7	**0.001**	**<0.001**	ns	ns	ns
			V2	14	21.7	5.9		18	21.2	5.1						
VAT [l]			V1	14	5.3	1.4	−0.4	18	6.8	2.5	−0.7	**0.024**	**<0.001**	ns	**0.042**	ns
			V2	14	4.9	1		18	6.1	2.3						
IHL (%)			V1	14	10.8	6.3	−3.8	17	14.5	11.3	−5.2	**0.003**	**<0.001**	ns	ns	ns
			V2	14	7	4.9		17	9.3	8.4						

Values are presented as mean (±SD), *p*-values of mean from *t*-test for analyzing the difference within each group are presented. Difference significant at *p* < 0.05. BMI = body mass index, TAT = total body fat, VAT = visceral adipose tissue, IHL = intra hepatic lipids. V1 = Visit 1, V2 = Visit 2. Δ = V2 − V1. ns = Not significant.

**Table 3:** Clinical parameter differences within and between diet groups.

												V1 vs V2		VLC vs LF		
Clinical Parameters				VLC			Δ	LF			Δ	VLC	LF	Δ	V1	V2
			Visit	*n*	Mean	±SD		*n*	Mean	±SD		*p*-Value	*p*-Value	*p*-Value	*p*-Value	*p*-Value
HbA1c (%)			V1	16	6.7	1	−0.6	20	6.2	0.6	−0.2	**<0.001**	ns	**0.001**	ns	ns
			V2	16	6.1	0.7		20	6.1	0.6						
CRP (mg/L)			V1	16	3.1	4.1	−1.3	20	3.1	2.7	−1.1	0.025 *	0.008 *	ns *	ns *	ns *
			V2	16	1.7	1.5		20	2	2.8						
T. CHO (mg/dL)			V1	16	200	39	−27	20	178	33	−31	**0.001**	**<0.001**	ns	ns	**0.042**
			V2	16	173	39		20	147	36						
HDL (mg/dL)			V1	16	56	16	−2	20	49	18	−4	ns	ns	ns	ns	ns
			V2	16	54	12		20	45	14						
LDL (mg/dL)			V1	16	127	36	−21	20	109	28	−26	**0.004**	**<0.001**	ns	ns	**0.035**
			V2	16	106	31		20	83	31						
TAG (mg/dL)			V1	16	133	65	−30	20	161	65	−35	**0.003**	**0.042**	ns	ns	ns
			V2	16	103	72		20	127	77						
SBP (mmHg)			V1	15	134	17	−9	19	134	21	−6	ns	ns	ns	ns	ns
			V2	15	126	10		19	128	19						
DBP (mmHg)			V1	15	85	11	−5	19	84	13	−6	ns	ns	ns	ns	ns
			V2	15	80	9		19	78	11						

Values are presented as mean (±SD), *p*-values of mean from t-test for analyzing differences within each group, which are presented (* = non-parametric test in the case of a missing normal distribution). TCHO= total cholesterol, HDL = high-density cholesterol, LDL= low-density cholesterol, TAG = triacylglycerides, CRP = c-reactive protein, HbA1c = glycated hemoglobin, SBP = systolic blood pressure in the right arm, DBP = diastolic blood pressure in the right arm, V1 = Visit 1, V2 = Visit 2 and Δ = V2 − V1.

**Table 4:** Macronutrient intake differences within and between diet groups.

				VLC			Δ	LF			Δ	VLC	LF	Δ	V1	V2
Macronutrient Intake			Visit	*n*	Mean	±SD		*n*	Mean	±SD		*p*-Value	*p*-Value	*p*-Value	*p*-Value	*p*-Value
Calories (kcal)			V1	14	2035	663	−717	15	2022	790	−1098	**<0.001**	**<0.001**	**<0.001**	ns	**0.001**
			V2	14	1318	220		15	925	61						
Protein (g/kg bw)			V1	14	0.95	0.15	0.21	15	0.94	0.2	−0.26	**0.002**	**<0.001**	**<0.001**	ns	**<0.001**
			V2	14	1.16	0.26		15	0.67	0.18						
Carbohydrate (g)			V1	14	196	69	−157	15	203	93	−85	**<0.001**	**0.006**	**0.005**	ns	**<0.001**
			V2	14	39	10		15	118	11						
Fat (%)			V1	14	40	9	14	15	38	6	−16	ns	**<0.001**	**<0.001**	ns	**<0.001**
			V2	14	54	6		15	21	2						

Values are presented as mean (±SD), *p*-values of mean from *t*-test for analyzing difference within each group are presented. Difference significant at *p* < 0.05. V1 = Visit 1, V2 = Visit 2 and Δ = V2 − V1. Macronutrients (and energy) *p*-value was based on g per kg BW calculations. Results are shown as mean ± SD.

**Table 5:** Differences within diet groups and diet interaction effect on FMD.

				VLC			Δ	LF			Δ	VLC	LF	Visit	Visit x Time
Endothelial Function			Visit	*n*	Mean	±SD		*n*	Mean	±SD		*p*-Value	*p*-Value	*p*-Value	*p*-Value
FMD (%)			V1	16	5.74	4.06	−0.26	20	4.32	3.5	2.27	ns	**0.024**	ns	**0.034**
			V2	16	5.48	3.21		20	6.59	4.69					

Values are presented as mean (±SD), *p*-values of mean from *t*-test for analyzing differences within each group are presented. FMD was calculated using ANOVA for repeated measurements adjusted for kilocalories intake changes per kg BW. Difference significant at *p* < 0.05. FMD = flow mediated dilation V1 = Visit 1, V2 = Visit 2 and Δ = V2 − V1.

## Supplementary Materials

The following are available online at http://www.mdpi.com/2072-6643/10/12/1859/s1, Table S1: Nutritional table of MODIFAST^®^ drink vanilla flavor.
