# Supplementary material for: Acute Endothelial Benefits of Fat Restriction over Carbohydrate Restriction in Type 2 Diabetes Mellitus: Beyond Carbs and Fats
Source: Nutrients. 2018 Dec 1;10(12):1859. doi: 10.3390/nu10121859 (PMC6316067; doi:10.3390/nu10121859)
Supplement: Supplementary file 1 [file nutrients-10-01859-s001.pdf]

Supplementary

Supplement Table S1. Nutritional table of MODIFAST® drink vanilla flavor.

	100g Powder	daily ratio (220 g)	Portion (55 g)	reference value ♂	♀	% RDA(220 g) ♂	f♀
**Energy (kJ)**	1652 kJ	3634 kJ	909 kJ				
**Energy (kcal)**	**392**	**862**	**216**	**2000**	**2500**	**43.1**	**34.5**
**Protein (g)**	**24.8**	**54.6**	**13.6**	**50**	**60**	**109.2**	**91**
**Carbohydrate (g)**	**50.6**	**111.3**	**27.8**	**270**	**340**	**41.2**	**32.7**
(Sugar) (g)	13.8	30.4	7.6				
(Lactose) (g)	8.4	18.5	4.6				
**Fat (g)**	**9**	**19.8**	**4.9**	**70**	**80**	**28.3**	**24.8**
(SFA) (g)	3.6	7.9	2	20	30	39.5	26.3
(MUFA) (g)	1.2	2.6	0.66				
(PUFA) (g)	2.1	4.6	1.2				
Linoleic acid (g)	2.1	4.6	1.2				
**Fiber (g)**	**4.6**	**10.1**	**2.5**	**30**	**30**	**33.7**	**33.7**
**Natrium (g)**	**0.4**	**0.88**	**0.22**	**0.55**	**0.55**	**160**	**160**
**Vitamins**							
Vitamin A (µg)	382	840	212	800	1000	105	84
Vitamin D (µg)	3	6.6	1.7	20	20	33	33
Vitamin E (mg)	7	15	3.9	12	14	125	107.1
Vitamin B1 (mg)	1.2	2.6	0.7	1	1.2	260	216.7
Vitamin B2 (mg)	1.2	2.6	0.7	1.2	1.4	216.7	185.7
Vitamin B6 (mg)	0.9	2	0.5	1.2	1.5	166.7	133.3
Vitamin B12 (µg)	0.99	2.2	0.55	3	3	73.3	73.3
Vitamin C (mg)	27	59	15				
Biotin (µg)	14	31	7.8	30-60	30-60	59	59
Folic acid (µg)	140	308	78	300	300	102.7	102.7
Niacine (mg)	9.4	21	5	13	16	161.5	131.3
Pantothenic acid (mg)	2.8	6.2	1.5	6	6	103.3	103.3
**Mineral Salts**							
Calcium (mg)	455	1001	252	1000	1000	100.1	100.1
Magnesium (mg)	103	227	57	325	325	69.8	69.8
Phosphorus (mg)	391	860	217	700	700	122.9	122.9
Potassium (mg)	1410	3102	783	2000	2000	155.1	155.1
**Trace Elements**							
Iron (mg)	7.8	17	4.3	10	15	170	113.3
Copper (mg)	0.67	1.5	0.37	7	10	21.4	15
Manganese (mg)	0.64	1.4	0.35	3.5	3.5	40	40
Zinc (mg)	6.6	15	3.7	7	10	214.3	150
Selenium (µg)	36	79	20	50	50	158	158
Iodine (µg)	125	275	69	200	200	137.5	137.5

Nutritional table of MODIFAST® drink vanilla flavor. Values are presented as means. The daily intake (%) 220 g formula (4 bags of 55 g) is compared with the recommended nutritional intake [1]. SFA: Saturated fatty acids, MUFA: Monounsaturated fatty acids, PUFA: Polyunsaturated fatty acids.

1. Wolfram, G. New reference values for nutrient intake in Germany, Austria and Switzerland (DACH-Reference Values). *Forum. Nutr.* **2003**, *56*, 95–97.
